# Anthrax Cases Associated with Animal-Hair Shaving Brushes

**DOI:** 10.3201/eid2305.161554

**Published:** 2017-05

**Authors:** Christine M. Szablewski, Kate Hendricks, William A. Bower, Sean V. Shadomy, Nathaniel Hupert

**Affiliations:** Centers for Disease Control and Prevention, Atlanta, Georgia, USA (C.M. Szablewski, K. Hendricks, W.A. Bower, S.V. Shadomy, N. Hupert);; Weill Cornell Medicine, New York, New York, USA (N. Hupert)

**Keywords:** Anthrax, Bacillus anthracis, fomites, warfare, disinfection, zoonoses, animal hair, shaving brushes, bacteria

## Abstract

During the First World War, anthrax cases in the United States and England increased greatly and seemed to be associated with use of new shaving brushes. Further investigation revealed that the source material and origin of shaving brushes had changed during the war. Cheap brushes of imported horsehair were being made to look like the preferred badger-hair brushes. Unfortunately, some of these brushes were not effectively disinfected and brought with them a nasty stowaway: *Bacillus anthracis*. A review of outbreak summaries, surveillance data, and case reports indicated that these cases originated from the use of ineffectively disinfected animal-hair shaving brushes. This historical information is relevant to current public health practice because renewed interest in vintage and animal-hair shaving brushes has been seen in popular culture. This information should help healthcare providers and public health officials answer questions on this topic.

“Hopefully someone gave you a badger hair brush during the holidays…” begins a modern-era advertisement for a purveyor of high-end shaving supplies. Since the turn of the 21st century, there has been a resurgence of interest in luxury-brand, animal-hair shaving brushes, evocative of an idyllic premodern esthetic. In the spring of 2017, a Google search for “badger shaving brush shopping” produced  ≈1.8 million hits; the same search limiting results through 2000 produced only ≈100 hits. But this luxury comes with a footnote, regarding an era when the sale of improperly prepared animal-hair shaving brushes caused dozens of sometimes fatal cases of cutaneous anthrax.

In 1915, British military officials began investigating cutaneous anthrax appearing on the heads and necks of soldiers newly recruited to serve in the Great War (*1*). Although the outbreak was initially attributed to the “diabolical tactics of the enemy,” officials soon realized that the source of the problem lay closer to home: low-cost shaving brushes that were being supplied to the troops (*2*). We describe this outbreak on the basis of 3 sources of published data—outbreak summaries, surveillance data from the United States, and descriptions of individual cases—and discuss current risk.

Our first data source was early outbreak summaries from Europe and the United States. During 1915–1924, numerous shaving brush–associated cases were reported from the United States and England; 149 cases occurred in members of the US military; 28, in the British military; 17, in American civilians; and 50, in British civilians (*3*,*4*).

Our second data source was anthrax surveillance from the United States (Figure). Nationwide surveillance data for 1919–1924 (although noted at the time to be incomplete) suggest that contaminated shaving brushes accounted for at least 10% of all anthrax cases (*5**,**6*). The situation was worse in New York City (NY, USA), where during this period up to half of anthrax cases were linked to shaving brushes (*10*).

**Figure:**
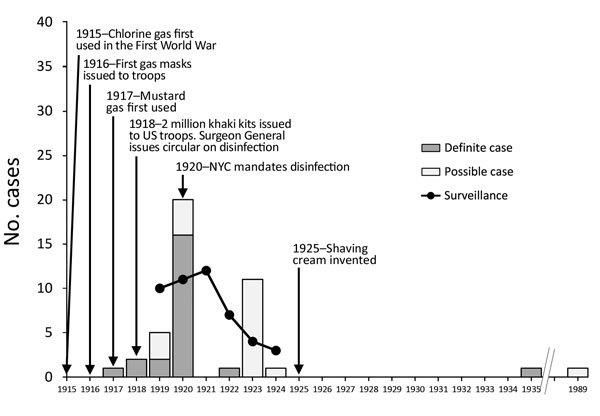
Timeline of use of shaving brushes and anthrax, 1915—1989. Case totals for the United States were reported in 1924 and 1930 and included 2 cases for 1927 through mid-1929, but the exact year of occurrence was unspecified (*5**,**6*). Data for English-language case descriptions were obtained from a systematic review of systemic anthrax cases published during 1880–2013 (*7*). Individual cases were reported from the United States, with the following exceptions: 1917, 1 definite case from England; 1918, 2 definite cases from Canada; 1920, 1 definite case from England; 1924, 1 possible case from South Africa; 1935, 1 definite case from Trinidad (*8*); and 1989, 1 possible case from India (*9*).

Our third data source was our recent review of systemic anthrax cases that were published in the English-language literature during 1880–2013 (Figure) (*7*). A total of 43 cases with anthrax definitely or possibly associated with shaving brushes were individually described in case reports, case series, or line lists during 1917–1989. Of these 43 cases, 20 (47%) were “possibly associated” on the basis of circumstantial evidence of usage of a recently purchased shaving brush and the absence of alternative exposure sources; the remainder were “definitely associated.” Of the individually described cases, 37% died; 85% of the survivors and 56% of those who died had been given antiserum. Most (84%) of the case-patients were from the United States. Although most shaving-associated anthrax cases occurred during 1917–1923, another 2 cases occurred well outside the outbreak period. The first was a cutaneous case in a patient from Trinidad in 1935, confirmed by both culture and guinea pig inoculation from a new goat-hair shaving brush (*8*). The second was a meningitis case in a patient from India in 1989, thought to be linked to a ritual shaving of the head the day before symptom onset (*9*). The age of the shaving brush was described for 25 of the 43 cases; 76% of the brushes were new and another 16% were <2 months old.

We are now able to explain the etiology and denouement of this mini epidemic. The First World War seems to have changed the demand for and the type, source, and treatment of hair used in shaving brushes (Figure). The use of chlorine gas in 1915 and mustard gas in 1917 led to the provision of 2 million “khaki kits” with safety razors to American troops in 1918 because it was believed that gas masks would be more effective on clean-shaven soldiers. Before the war, shaving brushes were generally made from hair from badgers, horses, or boars, but badger-hair brushes were the most popular because of their ability to hold water. However, with the wartime disruption of commerce, badger hair from Russia—then its main exporter—became difficult to acquire. In response, imitation “badger” brushes made from horsehair from Russia, China, or Japan appeared in the United States. Before the war, bundles of hair used to make shaving brushes were cleaned and disinfected in France or Germany while en route to the United States. During the war, however, the bundles were shipped directly to the United States (*11*).

Anthrax risk during 1914–1917 seems to have varied by brush color and country of origin. Cases were more likely to be associated with light- than with dark-colored brushes, and brushes from horsehair from Japan were considered to be particularly risky. Public health officials investigating these outbreaks at the time speculated that at least some of these manufacturers used the hair as received, assuming it was already disinfected (*2*). They also speculated that high-temperature disinfection may have been avoided for brushes made from light-colored hair out of concern that this treatment might diminish their resemblance to badger hair. Thus, light-colored brushes may not have been as effectively disinfected as their dark-colored or darkly dyed counterparts (*12*).

At least in New York City, a “smoking brush” was easy to find. In 1921, Bellevue researchers described testing shaving brushes recently purchased from New York City street vendors; they were able to confirm *B. anthracis* by guinea pig inoculation for 8% and to culture “anthracoid” bacilli from another 78%. Given the high proportion of brushes that seemed to be contaminated, these reviewers concluded that the only reason there weren’t more cases was “man’s relatively high degree of immunity to anthrax” (*3*).

After health officials determined that inadequate disinfection of shaving brushes was the reason for the outbreak, they enacted a series of control measures. These included a 1918 Surgeon General report publicizing a method for disinfecting brush hair, followed by a slew of edicts in 1920 by the New York City Board of Health, which described a method for disinfection, required all brushes for grooming (shaving, tooth, hair, nail, or other brush for human use) to be disinfected by use of this method, mandated labeling with both the manufacturer’s name and the word “sterilized,” and restricted sales to “sterilized” brushes (*5*,*13*).

Today, anthrax is rarely seen in the United States or the United Kingdom or mentioned outside the realm of bioterrorism preparedness and response. However, anthrax remains a reportable medical condition, and a search of the ProMED outbreak monitoring Web service suggests the international scope of the problem; in 2015, at least 400 human anthrax cases were reported worldwide. In the outbreak we describe, most cases occurred in American military or civilians during 1919–1923 and were associated with new imitation “badger-hair” brushes of equine origin. The equine connection is not surprising; research has shown that herbivores, such as horses, are more susceptible to anthrax than omnivores, such as badgers and pigs (*14*), and contemporaneous information on anthrax in US livestock mentioned that horses were more frequently affected than pigs (*15*). It is possible that hair destined for shaving brushes originating from the new sources across the Pacific was harvested from horses that had died of anthrax and then bypassed the cleaning and disinfection steps that had been in place before the war. 

Although the risk of acquiring anthrax from a shaving brush has been low since the mid-1920s, this article serves to remind those interested in a return to natural grooming that use of untreated hair from horses, pigs, badgers, or other animals poses a potential, and perhaps hypothetical, risk of inoculating anthrax spores into the abrasions and minor lacerations caused by shaving razors. Therefore, we emphasize the following points:

• Because of modern decontamination and import regulations (*16*,*17*), new animal-hair brushes are unlikely to be a source of anthrax.

• Risk from brushes manufactured in the United States after 1930 and well-used (even vintage) brushes would seem to be extremely low.

• We do not recommend trying to disinfect vintage brushes at home because the risks associated with various combinations of steam, pressure, and formaldehyde are likely to outweigh possible benefits.

This brief communication describes the history of anthrax and animal-hair shaving brushes. It should provide useful information for healthcare providers and public health officials answering questions on this topic.
